# The Unity of Disease Analytically and Synthetically Proved: With Facts and Cases Subversive of the Received Practice of Physic

**Published:** 1839-10

**Authors:** 


ART. II. — *The Unity of Disease analytically and synthetically proved: with Facts and Cases subversive of the received Practice of Physic.* By SAMUEL DICKSON, M.D.—*London*, 1839, pp. 200.

WE noticed in a previous number (*Brit. and For. Med. Rev.* vol. III. p. 469,) a remarkably silly book, of which the volume before us is an amplification. Our remarks, it appears, have been read by Dr. Dickson. Those which he regards as the most vulnerable he very feebly attacks; others, he wisely allows to pass without comment. We should have taken no notice of the present production but that when such pseudo-regenerators of medical science repeatedly attempt to puff themselves into an unworthy notoriety we think it right to abate somewhat the self-glorification of such pompous gentlemen. Did we believe that to ascertain truth was their object, we should have the additional desire to afford them a light upon the exceedingly dark path which they are taking in pursuit of it. For the doctrine of disease propounded by Dr. Dickson, and for its refutation, we refer to our former article, merely observing on this occasion, that whatever is new in the said doctrine is as silly as if the identity of diseases were attempted to be shown from the fact that mankind are always more or less wet, more or less heavy, more or less anything which may be said of the entire organism. Assuming for one moment that the author may wish to attain that important something which is said to lie at the bottom of a well—a very deep well, we fear, it must prove to his centripetal powers —we should recommend to his careful consideration the following advice of a man much used to close and continued reflection : " Whether you are reflecting with yourself or reasoning with another, make it a rule to ask yourself the precise meaning of the word on which the point in question appears to turn. By this means, and scarcely without it, you will at length acquire a facility in detecting the *quid pro quo*." Let him then take the words "cause," " health," "fever," and subject them to rigid definitions, reading afterwards the article already referred to in the Third Volume of this Journal. Having done these things in a proper spirit, we feel satisfied that, even if his great dedicatee, " *William Lord Viscount Melbourne*," should demand it, Dr. Dickson will not venture to publish a second edition.

That such of our readers as have not seen either our previous notice or Dr. Dickson's book, may not think our present commentary extravagant, we shall quote the author's own exposition of his doctrine in his concluding remarks.

"We have proved, we hope, to the satisfaction of all but the prejudiced and the interested—

"1. That the phenomena of perfect health consist in a regular series of alternate actions—each embracing a special portion of time.

"2. That disease, under all its modifications, is a simple exaggeration or diminution of the same actions ; and being universally alternative with a comparative state of health, strictly speaking, resolves itself into fever, remittent or intermittent, chronic or acute; every kind of structural lesion or disorganization, from the caries of a tooth, to the plumonary decomposition of phthisis, and that state of knee which is termed white swelling, being merely developments in its course (Tooth-consumption,—Lung-consumption,—Knee-consumption.)"

" 3. That the tendency to disorganization, usually denominated acute or inflammatory, differs from the chronic or scrofulous in the mere amount of temperature and action : the former being more remarkably characterized by excess of both, and consequently exhibiting a more rapid progress to decomposition or cure; while the latter approaches its respective terminations, by more subdued, and consequently slower and less obvious alternations of the same action and temperature. The slow and rapid caries of a tooth vary [varies] in nothing, from the chronic and "galloping" consumptions, except in the difference of tissue involved, and the degree of danger to life, arising out of the nature of the respective offices of each."

"Disease, thus simplified, will be found to be amenable to a principle of treatment equally simple. Partaking of the nature of ague, throughout all its modifications, it will be best met by a practice in accordance with the proper treatment of this." (pp. 188-9.)

